# Impact of Intersecting Systems of Oppression on Diabetic Retinopathy Screening Among Those Who Identify as Women of Low Socioeconomic Status: Protocol for a Convergent Mixed Methods Study

**DOI:** 10.2196/23492

**Published:** 2021-03-05

**Authors:** Aleksandra Stanimirovic, Troy Francis, Anna Cooper Reed, Sonia Meerai, Olivera Sutakovic, Rebecca Merritt, Michael Brent, Valeria Rac

**Affiliations:** 1 Program for Health System and Technology Evaluation Toronto General Hospital Research Institute University Health Network Toronto, ON Canada; 2 Ted Rogers Centre for Heart Research at Peter Munk Cardiac Centre Toronto General Hospital Research Institute University Health Network Toronto, ON Canada; 3 Institute of Health Policy, Management and Evaluation University of Toronto Toronto, ON Canada; 4 Diabetes Action Canada CIHR SPOR Network Toronto, ON Canada; 5 Toronto Health Economics and Technology Assessment Collaborative Toronto General Hospital Research Institute University Health Network Toronto, ON Canada; 6 Gender, Feminist & Women Studies Faculty of Graduate Studies York University Toronto, ON Canada; 7 Lyle S Hallman Faculty of Social Work Wilfrid Laurier University Brantford, ON Canada; 8 Department of Ophthalmology Toronto Western Hospital Toronto, ON Canada; 9 South Riverdale Community Health Centre Toronto, ON Canada

**Keywords:** gender, screening, diabetes, diabetic retinopathy, blindness, technology, tele-retina screening, health equity, intersectionality theory

## Abstract

**Background:**

By 2025, 5 million Canadians will be diagnosed with diabetes, and women from lower socioeconomic groups will likely account for most new diagnoses. Diabetic retinopathy is a primary vision complication of diabetes and a leading cause of blindness among adults, with 26% prevalence among women. Tele-retina is a branch of telemedicine that delivers eye care remotely. Screening for diabetic retinopathy has great potential to reduce the incidence of blindness, yet there is an adverse association among screening, income, and gender.

**Objective:**

We aim to explore gender disparity in the provision of tele-retina program services for diabetic retinopathy screening in a cohort of women of low socioeconomic status (SES) receiving services in South Riverdale Community Health Centre (SRCHC) between 2014 and 2019.

**Methods:**

Using a convergent mixed methods design, we want to understand patients’, providers’, administrators’, and decision makers’ perceptions of the facilitators and barriers associated with the implementation and adoption of tele-retina. Multivariate logistic regression will be utilized to assess the association among client characteristics, referral source, and diabetic retinopathy screening. Guided by a grounded theory approach, systematic coding of data and thematic analysis will be utilized to identify key facilitators and barriers to the implementation and adoption of tele-retina.

**Results:**

For the quantitative component, we anticipate a cohort of 2500 patients, and we expect to collect data on the overall patterns of tele-retina program use, including descriptions of program utilization rates (such as data on received and completed diabetic retinopathy screening referrals) along the landscape of patient populations receiving these services. For the qualitative component, we plan to interview up to 21 patients and 14 providers, administrators, and decision makers, and to conduct up to 14 hours of observations alongside review of relevant documents. The interview guide is being developed in collaboration with our patient partners. Through the use of mixed methods research, the inquiry will be approached from different perspectives. Mixed methods will guide us in combining the rich subjective insights on complex realities from qualitative inquiry with the standard generalizable data that will be generated through quantitative research. The study is under review by the University Health Network Research Ethics Board (19-5628). We expect to begin recruitment in winter 2021.

**Conclusions:**

In Ontario, the screening rate for diabetic retinopathy among low income groups remains below 65%. Understanding the facilitators and barriers to diabetic retinopathy screening may be a prerequisite in the development of a successful screening program. This study is the first Ontario study to focus on diabetic retinopathy screening practices in women of low SES, with the aim to improve their health outcomes and revolutionize access to quality care.

**International Registered Report Identifier (IRRID):**

PRR1-10.2196/23492

## Introduction

### Social and Economic Impacts of Diabetes and Its Complications

Diabetes is a significant public health burden, affecting 382 million people worldwide [1]. In Canada, the prevalence was estimated at 3.4 million (9.3%) in 2015 and is expected to increase to 5 million (12.1%) by 2025 [2]. Diabetic retinopathy is the primary vision complication caused by diabetes [3] and is the leading cause of new cases of blindness in adults aged 20 to 65 years [4]. The prevalence of diabetic retinopathy in Canada ranges from 20% to 30% [4]. Over a million people from Ontario were affected by diabetic retinopathy in 2016 [5]. Among Canadian adults, 5.7% have visual impairments with a variation in the provincial prevalence of visual impairment from 2.4% in Manitoba to a staggering 10.9% in Newfoundland and Labrador [6]. Lower income and type 2 diabetes have been shown to be associated with increased odds of visual impairment [6].

One-third of adult diabetic patients did not receive an eye examination for diabetic retinopathy within 2 years [5], and more specifically, 25.3% of people with diabetes over the age of 60 years had not seen an eye care provider in the last year [7]. The prevalence of vision loss in Canada is expected to increase nearly 30% in the next decade [8]. The financial implication of vision loss in Canada in 2007 was estimated to be CAD $15.8 billion per annum, with CAD $8.6 billion (54.6%) associated with direct health system expenditure; CAD $4.4 billion (28.0%) associated with productivity loss resulting from lower employment, higher absenteeism, and premature death of Canadians with vision loss; CAD $1.8 billion (11.1%) associated with costs to society created by market inefficiency from transfers including welfare payments and taxation forgone; CAD $0.7 billion (4.4%) associated with the value for the care of people with vision loss; and CAD $305 million (1.9%) associated with other indirect costs such as aids, home modifications, and funeral costs [9]. The value of the lost well-being (inclusive of disability and premature death) was estimated at CAD $11.7 billion. In per capita terms, this adds to a financial cost of CAD $19,370 per person with vision loss per annum, and considering the value of lost well-being, the cost is CAD $33,704 per person per annum [9]. The Canadian National Institute for the Blind (CNIB) estimated costs of associated complications of vision loss are as follows: falls, CAD $25.8 million; depression, CAD $175.2 million; hip fractures, CAD $101.7 million; and nursing home admission, CAD $713.6 million [10]. The National Coalition for Vision Health noted that health care costs for vision loss in Canada have been projected to increase to CAD $30.3 billion per year by 2032 [11].

### Health Disparities in Diabetes and Its Complications and Comorbidities

Health disparities in diabetes and its complications exist globally [12]. In Canada, ethnic minorities and Indigenous populations have a higher prevalence of diabetes than nonminority populations [13]. Diabetes appears to be more common among men than women. Socioeconomic status (SES) is inversely related to the prevalence of diabetes, but income-related disparities are greater among women [13]. In comparison to men with diabetes, women were more likely to be in the lowest income quintiles than the highest [14]. The odds ratio of developing diabetes doubles in men and almost triples in women in the lowest income category compared with those in the highest income category [15], and among Aboriginal Canadians, two-thirds (66.6%) of diagnosed individuals are women [16]. The Project for an Ontario Women’s Health Evidence–based Report (POWER) study found that women of lower SES with diabetes had worse health and functional statuses than men and stressed the importance of addressing gender differences, which may interfere with diabetes self-care among the general diabetic population [14]. In 2016, 1.8 million Canadian males and 2.2 million females aged 45 to 85 years experienced vision loss. Prevalence increased from 8.7% to 16.9% between 2011 and 2016 [17]. Prevalence proportions increased with age but decreased exponentially with the severity of impairment, and vision loss remains more common among females [17].

An adverse association between screening and income has been found previously. A published report indicated that women of lower SES may not be screened for breast, lung, and colorectal cancers [3]. In fact, they may not have symptoms recognized early or receive the most effective treatment. Similar to cancer screening, diabetic retinopathy screening is essential for the early detection and treatment of diabetes-related visual impairments and blindness [18]. Yet, it is commonly underutilized among women of lower SES [18]. Because diabetic morbidity and mortality are associated with low SES, the need to address socioeconomic barriers for women must take precedence over simply ensuring the provision of diabetes medical management. This is the first Ontario study to focus on diabetic retinopathy screening practices in women of low SES, with great potential to improve their health outcomes and access to quality care.

### Tele-Retina Screening for Diabetic Retinopathy

There are disparities in eye care utilization among community-dwelling Canadians, where eye care utilization is defined as the self-report of a visit to an optometrist or ophthalmologist in the past 12 months [7]. Of concern, 25.3% of people with diabetes above the age of 60 years had not seen an eye care provider in the last year. Men in comparison to women and people with lower income (linear trend *P*<.05) were less likely to use eye care [7].

Tele-retina is one of the diabetic retinopathy screening modalities. It is focused on reducing eye care disparities that lead to avoidable vision loss. Tele-retina is a branch of telemedicine that delivers eye care remotely. Retinal images and data are collected and transferred via telecommunication technology to eye specialists [19]. In many developing countries, tele-retina has been utilized to provide quality eye care to the underserved urban population and the unserved remote rural population [19]. Alternatives to in-person examinations, such as tele-retina, can triage patients to proper levels of care and reduce barriers to specialized eye care [19]. Screening and detection of diabetic retinopathy are important to reduce the incidence of blindness, as they can detect early sight-threatening lesions, which can be treated effectively. Factors contributing to patients’ missed opportunities in access to timely treatment can include limited number of specialists and challenges related to time and travel.

The eye care pathway does not end with diabetic retinopathy screening. Individuals who are screened and those who remain unscreened and develop severe vision loss have access to comprehensive vision rehabilitation services. In Canada, comprehensive vision rehabilitation represents a multidisciplinary pathway that encompasses the full spectrum of a patient’s rehabilitation journey after vision loss, from initial assessment through intensive rehabilitation therapy [19].

Of note, in this study, tele-retina was utilized in an urban setting. It is also being utilized in rural settings such as First Nations reserves. It has the potential to scale to many rural communities that are underserviced with respect to diabetic retinopathy screening and could be used as a strategy in conjunction with the Medical Mobile Care Unit (known as the CNIB Eye Van). The CNIB Eye Van is a fully equipped medical mobile eye care clinic that travels (with an ophthalmologist on board) to patients in Northern Ontario, from March through October each year [20,21]. The CNIB Eye Van was cancelled in 2020 owing to the COVID-19 pandemic, leaving patients without care. A tele-retina program is a viable option that could address the needs of these patients.

### Conceptual Frameworks

The Conceptual Social Determinants of Health framework adapted from the World Health Organization, Danaher framework, and Multi-Construct Intersectionality framework (Synergies of Oppression) will provide theoretical insights into the potential facilitators and barriers of the implementation and adoption of tele-retina screening for diabetic retinopathy. The frameworks will be used as analytical lenses to assess the system-, organizational-, and patient/provider-level causal factors of the tele-retina program. This approach will provide in-depth insights into the experiences, impacts, and outcomes of tele-retina screening for diabetic retinopathy among women in low socioeconomic groups.

#### Social Determinants of Health

Social determinants of health are conceptualized in a manner that takes into account how environmental and material conditions further increase the risk for marginalized populations at the intersection of identities such as race, age, gender, and income [22-24]. Social determinants of health in the context of mobile health screening among marginalized populations take into consideration environmental stressors that an individual may be exposed to, including but not limited to toxins, dwelling living conditions, access to education, food, employment, the role of a globalized economy and its effects, and current social exclusionary measures exacerbated through limited access to basic needs [22-24].

Integrating a socioeconomic approach provides an integrative synergistic framework in further understanding the material effects and experiences of diabetic retinopathy screening among women with low SES. Embedded within the framework is a social, economic, and political system approach for uncovering health inequities that are heavily influenced by these systems [23-25]. In this context, social determinants of health are elevated to include systems that perpetuate oppressions in relation to these material effects, such as poverty and inaccessibility to housing, food, and employment, which further compounds the overall health and well-being of women with low SES [23-26].

#### Health Equity

**Figure 1:**
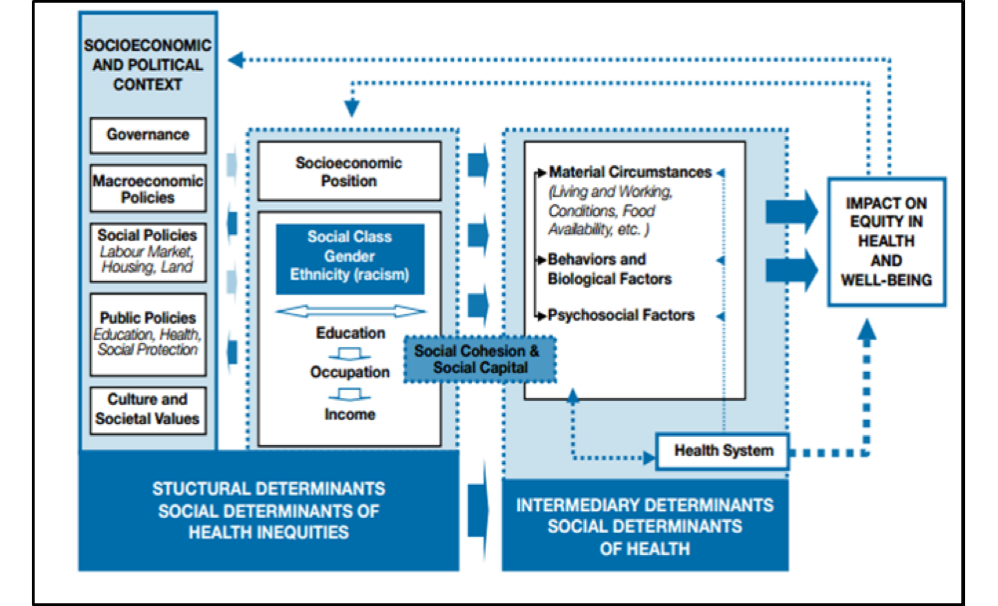
Conceptual Social Determinants of Health framework (adapted from the World Health Organization).

**Figure 2:**
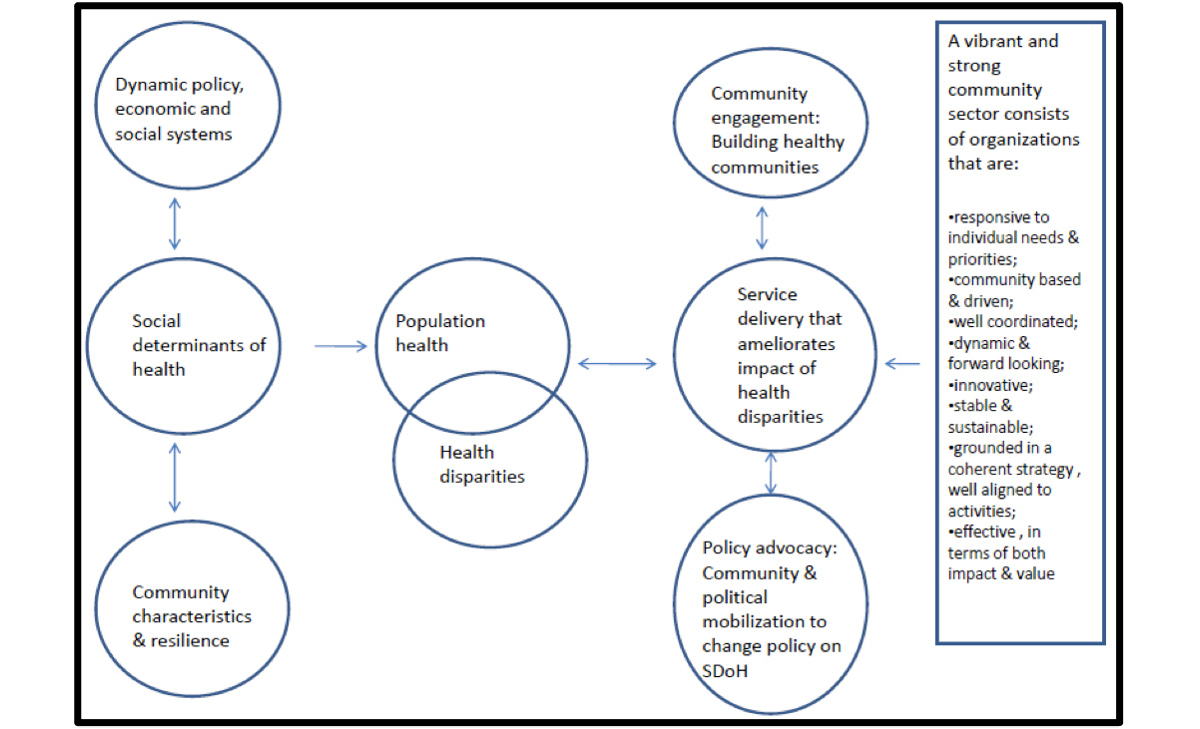
Danaher framework. SDoH: Social Determinants of Health.

Health equity outcomes are directly correlated with the distribution of resources within any particular context. Women of low SES with multiple health conditions and limited access to resources have decreased access to achieving health equity. When deconstructing health equity, the construct of power relations within social, economic, and political systems is taken into consideration, as it determines the success rates for health- and well-being–specific interventions, including but not limited to promotion of health, interventions, and evaluation of the effectiveness of health-specific programs [22]. In considering the Social Determinants of Health framework, health equity, specifically the concept of power relations with a distribution of resources, is integral to understanding how successful health promotion and intervention will be sustainable for marginalized populations [22]. Figure 1 illustrates the synergy of adapting the Social Determinants of Health framework with health equity [22], and Figure 2 illustrates the Danaher framework (2011), which integrates the role of community in health policy advocacy for addressing health disparities [27].

#### Intersectionality

**Figure 3:**
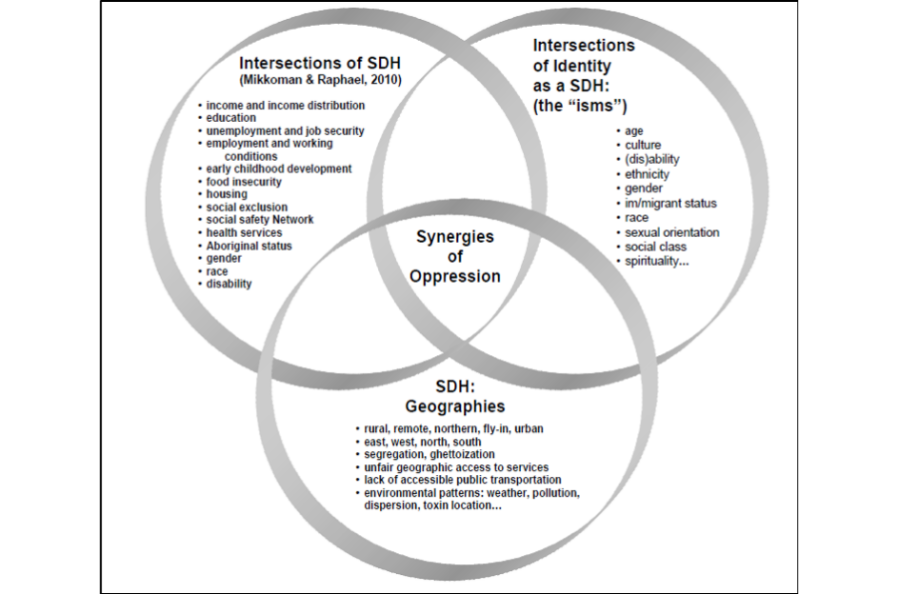
Multiconstruct intersectionality framework (Synergies of Oppression) for addressing social determinants of health (SDH) inequities (adapted from McGibbon & McPherson [28]).

Intersectionality is a theoretical and pragmatic tool within health service and system research to further nuance the complexities of the impact of health promotion and intervention among marginalized populations. Originally developed by Black feminist scholar Kimberle Crenshaw (1989), it has been adapted and interrogated to be applied in many other contexts within the sciences, social sciences, and humanities [23]. Marginality in relation to an intersectional approach takes into consideration that identity markers, such as age, race, gender, disability, and SES, are not viewed as separate and are rather interwoven with the outcomes involving how power relations within social, economic, and political systems further create health inequities through inaccessibility to resources for health promotion and intervention [23-26]. Intersectionality is taken up in this conceptual framing of an intersectional categorical axis where social determinants of health, systems of oppressions, and environmental factors are integrated [23-26]. Figure 3 illustrates these categories/framings within the context of the synergy of oppressions, specifically the outcomes of increased marginalization through oppressions and intersections of social determinants of health. This synergy is also compounded with access to health equity [26,28].

For the purpose of this study, social determinants of health, health equity, and an intersectional approach to analysis are integral for understanding the impacts and material effects of diabetic retinopathy screening among women of low SES. This adapted framework provides an in-depth approach to understand how power relations within social, economic, and political systems impact community-based health screening programs for marginalized populations. Further, it integrates the individual standpoint based on intersectional identities and the environment.

### Program Description

**Figure 4:**
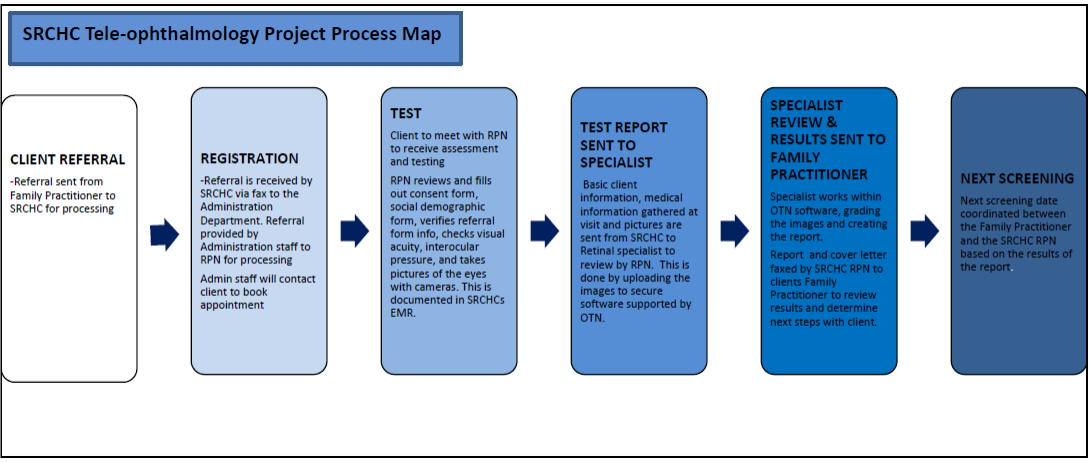
South Riverdale Community Health Centre (SRCHC) tele-retina project process map [30]. EMR: electronic medical records; OTN: Ontario Telemedicine Network; RPN: Registered Practical Nurse.

In 2014, South Riverdale Community Health Centre (SRCHC), in partnership with Dr Michael H Brent, Chief of Retina Services at the University of Toronto, received funding from Toronto Central Local Health Integration Network to develop a mobile screening program to assess the retinal health of individuals diagnosed with diabetes (Figure 4) [29]. This strategy is driven by the recognition that access to optometrists and ophthalmologists is difficult for individuals with diabetes who live in certain neighborhoods in Toronto.

The tele-retina program is offered to patients at no cost in partnership with primary care organizations with a population focus (Anishnawbe Health Toronto) or in low-income communities with high prevalence of diabetes and low diabetic retinopathy screening rates (Parkdale, Flemingdon Park Community Health Centre, Scarborough Academic Family Health Team, LAMP Community Health Centre, Unison Health, Community Services-Lawrence Heights Site, and Unison Health and Community Services sublocations [Bathurst-Finch, Jane-Trethewey, and Keele-Rogers]) [29,30].

As of December 2016, the Toronto Health Economics and Technology Assessment Collaborative has created a very strong collaboration with SRCHC where Toronto Health Economics and Technology Assessment assessed the cost-effectiveness of the screening program [31]. In 2019, findings from a cost-effectiveness study suggested that tele-retina is a more cost-effective means of screening for diabetic retinopathy than the standard of care screening in urban and rural underscreened communities, and this study represents a natural progression of the previous collaborative work.

## Methods

### Guidelines

This protocol has been developed in accordance with SPIRIT (Standard Protocol Items: Recommendations for Interventional Trials) guidelines [32].

### Aims

We aim to explore the gender disparity in the provision of tele-retina program services for diabetic retinopathy screening in a cohort of women of low SES receiving services in SRCHC between 2014 and 2019, including but not limited to the evaluation of the overall patterns of tele-retina program use, and to conduct a qualitative study of patients, providers, administrators, and decision makers in order to understand their perceptions regarding the facilitators and barriers associated with the implementation and adoption of the tele-retina program.

### Study Population

#### Quantitative Component

The population of interest includes individuals of low SES (income less than CAD $25,000) attending diabetic retinopathy screening services at various sites (listed in Table 1) from 2014 to 2019, who have a diagnosis of diabetes, are at high risk for remaining unscreened for diabetic retinopathy, and have limited access to eye care.

SRCHC annually serves a population of 10,000 individuals, of which about 13% are diagnosed with diabetes and should undergo diabetic retinopathy screening. Annually, close to 500 individuals diagnosed with diabetes and receiving services from SRCHC are screened for diabetic retinopathy. As the program has been in place since 2014, we anticipate the study cohort will consist of 2500 individuals (500 individuals screened for diabetic retinopathy per year × 5 years [from 2014 to 2019]).

**Table 1:** Breakdown of recruitment (qualitative component) based on participating sites.

Site	Number of patient interviews	Number of health care provider, administrator, and decision maker interviews	Number of hours of observation
Anishnawabe Health Toronto	2-3	1-2	1-2
Flemingdon Health Centre	2-3	1-2	1-2
LAMP Community Health Centre	2-3	1-2	1-2
Parkdale Community Health Centre	2-3	1-2	1-2
Scarborough Academic Family Health Team	2-3	1-2	1-2
South Riverdale Community Health Centre	2-3	1-2	1-2
Unison Health and Community Services	2-3	1-2	1-2

#### Qualitative Component

The population of interest includes those who identify as women of low SES who attend or decline to attend tele-retina screening for diabetic retinopathy within one of the identified sites. The study population also includes health care providers, administrators, and decision makers who are involved in the tele-retina screening program.

Within the qualitative component, in addition to interviewing providers, administrators, and decision makers, we will purposely select a subset of the population from the quantitative component, more specifically those identifying as women of low SES who attend or decline to attend tele-retina screening for diabetic retinopathy within one of the identified sites.

### Inclusion Criteria

Patients who are aged 18 years or above; identify as women of low SES (income less than CAD $25,000); are able and willing to provide verbal and/or written informed consent; and attend (complete or decline) tele-retina screening for diabetic retinopathy are considered for inclusion. Please note that there are no language requirements, as we will use a translation services for non-English speaking clients.

Health care providers who currently provide care and are part of the screening process within the tele-retina program are considered for inclusion.

Administrators who coordinate the tele-retina screening for diabetic retinopathy and decision makers who inform and/or are part of the decision-making process for tele-medicine–specific programming within community health centers in Ontario are considered for inclusion.

### Exclusion Criteria

Patients who are aged less than 18 years; do not identify as women of low SES (income less than CAD $25,000); and are unable or unwilling to provide verbal and/or written informed consent are excluded.

Health care providers who are not practicing health care providers in the tele-retina screening for diabetic retinopathy program and are unable or unwilling to provide verbal informed consent are excluded.

Administrators and/or decision makers who are unable or unwilling to provide verbal informed consent are excluded.

### Data Collection

#### Quantitative Component

In the retrospective cohort study, we will collect data on the overall patterns of tele-retina program use, including descriptions of program utilization rates (such as data on received and completed diabetic retinopathy screening referrals) along the landscape of patient populations receiving these services.

#### Qualitative Component

The qualitative component will entail collection of primary data sources to ensure rigor and data quality, including (1) nonparticipatory observations (ethnographic observations), (2) semistructured interviews, and (3) document review.

We anticipate a total of 7 to 14 hours of nonparticipatory observations (1-2 hours of observation per site) to observe how providers carry out their work on a daily basis, how administrators interact with providers, how patients and providers interact, and how patients interact with their environments. Field work provides excellent opportunities to identify and engage respondents for interviews and to collect grey literature. For all such field work, we will document activities, impressions, and interactions through field notes. Interviews lasting approximately 60 minutes will be guided by open-ended semistructured interview guides, and recorded and transcribed verbatim [33]. Interview guides will ensure that interactions among the researcher and participants remain focused and will be modified as new themes or issues arise, in line with the qualitative approach [34]. Textbox 1 provides a detailed description of ethnographic field work and semistructured interviews.

We will collect and review relevant documentary sources on the operation of the tele-retina program, as processes within organizations are frequently text based and may serve as a substitute for records of activity [35]. Documents (eg, scientific papers, conference reports, organizational histories, press releases, and news stories) may provide access to an accepted body of knowledge about the role, policy, and procedures of an organization [36]. Documents will be collected by searching for publicly available documents (eg, through organizational websites, citations, and database searches).

**Description of ethnographic field work and semistructured interviews per identified site.**

**Ethnographic fieldwork**

- Process and interaction between the patient and health care provider during the tele-retina screening process for diabetic retinopathy (7-14 hours in total)

**Semistructured interviews per identified site**

*Patients*

- 2-3 one-on-one semistructured interviews per site
- Patients who identify as women of low socioeconomic status
- Impact, experience, and outcomes of participating in the screening process

*Health care providers*

- 7-14 semistructured interviews
- Nurses, physicians, and/or ophthalmologists involved in the screening process
- Impact and experience of coordination, and delivery of the program

*Administrators/decision makers*

- Up to 14 semistructured interviews
- Health care administrators/decision makers
- Values and beliefs that inform the screening program
- Description of the coordination, delivery, funding, and policies that inform the screening program

### Analysis

#### Quantitative Data Analysis

Continuous variables will be described using measures of central tendency and dispersion, such as mean/median and standard deviation/interquartile range, with appropriate statistical methods, and compared using analysis of variance (ANOVA) or the Kruskal-Wallis test as appropriate. Categorical variables will be described using contingency tables and compared using the chi-square test. Multivariate logistic regression will be utilized to assess the association between client characteristics, screening referral sources, and diabetic retinopathy screening. Analyses will be conducted using SAS v 9.4 (SAS Institute).

#### Qualitative Data Analysis

Data analysis will be an iterative and inductive process using a grounded theory approach [37]. This will involve systematic coding of data and theme abstraction to identify key facilitators and barriers to tele-retina intervention across the sites to ensure that our findings will inform recommendations appropriate to their context. Thematic analysis of the interview transcripts, interview notes, and observation notes will occur in the following three stages: open coding (data reduction), axial coding (data display), and selective coding (conclusion drawing). Comparisons within and across the interview data will be conducted (constant comparison technique) [38]. Multiple readings will be used, and alternative explanations of the data will be explored [39,40] to develop the most plausible and robust interpretation of the findings in order to obtain a comprehensive understanding of the facilitators and barriers to diabetic retinopathy screening [41,42]. All final themes will be informed by continuous dialogue among the research team. This dialogue will facilitate self-reflection on how the analysis evolved to allow the qualitative lead to fully interrogate potential assumptions or biases reflected in the interpretation of the data [37]. Reliability of the findings will be strengthened by maintaining a chain of evidence throughout the study to ensure that the evolution of qualitative results can be followed by an external observer in order to ensure credibility of the data collection and analytical process. Data will be stored and managed electronically using the qualitative research software NVivo 11 (QSR International).

#### Convergent Analysis and Interpretation

We will utilize a convergent mixed methods design, which will combine and contrast the data collected in the quantitative and qualitative components in order to triangulate similarities and differences in the results of both research methods [43]. The findings will summarize and interpret to what extent the results from the two components converge, diverge, and produce a more complete understanding of the use of tele-retina services among women of low SES receiving care at SRCHC [43].

## Results

For the quantitative study, we anticipate a cohort of 2500 patients, which will provide descriptive information on patterns of use of the tele-retina program. In total, we plan to interview 14 to 21 patients and 7 to 14 providers, administrators, and decision makers, and to conduct 7 to 14 hours of observations in order to gain an understanding of the facilitators and barriers to diabetic retinopathy screening. Please note that the interview guide is being developed in collaboration with our patient partners. The study is under review by the University Health Network Research Ethics Board (19-5628). We responded to the first set of Research Ethics Board comments and are anticipating Research Ethics Board response. We expect to begin recruitment in winter 2021, as the tele-retina program has recently resumed at SRCHC.

This work is supported by Patient-Oriented Research Intercentre Trainee Internship in Diabetes and its Complications through Diabetes Action Canada–Canadian Institutes of Health Research funds and an unrestricted educational grant from Novartis Canada. The funding sources have no involvement in the study design; the collection, analysis, or interpretation of data; the writing of the manuscript; or the decision to submit the article for publication.

## Discussion

This protocol outlines a study designed to understand the critical facilitators and barriers of the delivery of diabetic retinopathy screening among vulnerable communities. With the increasing incidence and prevalence of diabetes worldwide, morbidity, mortality, and associated costs due to diabetes-related complications remain a growing public health concern [44]. Diabetic retinopathy represents a global epidemic, as 191 million individuals worldwide will be diagnosed by 2030 [44], and the disease burden remains concentrated among low-income groups [45,46]. Emerging evidence illustrates that often interventions aim to improve access to care, but may not be well adapted to vulnerable populations [47]. Understanding the facilitators and barriers of screening in this population will address the knowledge gap and assist in developing, implementing, and adopting effective, yet culturally sensitive, diabetic retinopathy screening interventions and thus carries promise in reducing the burden of blindness resulting from diabetic retinopathy. The findings should generate a deeper understanding of the ways in which system-level organizational interventions may improve access to screening for vulnerable populations and new knowledge with regard to improvements in the delivery of diabetic retinopathy screening interventions. Considering the widespread burden of diabetic retinopathy across the globe, the findings will be disseminated to ensure that strategies for the prevention and treatment of diabetic retinopathy are sensitive to vulnerable populations and can be implemented and adopted at the global level.

Considering that previous work has found tele-retina screening to be a more cost-effective alternative to standard care and that there is an increasing global burden of diabetic retinopathy, there is a need for improved access to care for vulnerable populations. National and international scaling and adoption of the tele-retina program to assist vulnerable populations may contribute to system-level cost saving.

## Abbreviations

CNIB: Canadian National Institute for the Blind
SES: socioeconomic status
SRCHC: South Riverdale Community Health Centre
